# Universal coverage of the first antenatal care visit but poor continuity of care across the maternal and newborn health continuum among Nepalese women: analysis of levels and correlates

**DOI:** 10.1186/s12992-021-00791-4

**Published:** 2021-12-11

**Authors:** Resham B Khatri, Rajendra Karkee, Jo Durham, Yibeltal Assefa

**Affiliations:** 1 School of Public Health, Faculty of Medicine, University of Queensland, Brisbane, Australia; 2 Health Social Science and Development Research Institute, Kathmandu, Nepal; 3 School of Public Health and Community Medicine, BP Koirala Institute of Health Sciences, Dharan, Nepal; 4 School of Public Health and Social Work, Queensland University of Technology, Brisbane, Australia

**Keywords:** antenatal care, (dis)continuity of care, essential interventions, institutional delivery, postnatal care, routine maternity care visits

## Abstract

**Background:**

Routine maternity care visits (MCVs) such as antenatal care (ANC), institutional delivery, and postnatal care (PNC) visits are crucial to utilisation of maternal and newborn health (MNH) interventions during pregnancy-postnatal period. In Nepal, however, not all women complete these routine MCVs. Therefore, this study examined the levels and correlates of (dis)continuity of MCVs across the antenatal-postnatal period.

**Methods:**

We conducted further analysis of the 2016 Nepal Demographic and Health Survey. A total of 1,978 women aged 15–49 years, who had live birth two years preceding the survey, were included in the analysis. The outcome variable was (dis)continuity of routine MCVs (at least four ANC visits, institutional delivery, and PNC visit) across the pathway of antennal through to postnatal period. Independent variables included several social determinants of health under structural, intermediary, and health system domains. Multinomial logistic regression was conducted to identify the correlates of routine MCVs. Relative risk ratios (RR) were reported with 95% confidence intervals at a significance level of *p*<0.05.

**Results:**

Approximately 41% of women completed all routine MCVs with a high proportion of discontinuation around childbirth. Women of disadvantaged ethnicities, from low wealth status, who were illiterate had higher RR of discontinuation of MCVs (compared to completion of all MCVs). Similarly, women who speak Bhojpuri, from remote provinces (Karnali and Sudurpaschim), who had a high birth order (≥4), who were involved in the agricultural sector, and who had unwanted last birth had a higher RR of discontinuation of MCVs. Women discontinued routine MCVs if they had poor awareness of health mother-groups and perceived the problem of not having female providers.

**Conclusions:**

Routine monitoring using composite coverage indicators is required to track the levels of (dis)continuity of routine MCVs at the maternity care continuum. Strategies such as raising awareness on the importance of maternity care, care provision from female health workers could potentially improve the completion of MCVs. In addition, policy and programmes for continuity of maternity care are needed to focus on women with socioeconomic and ethnic disadvantages and from remote provinces.

**Supplementary Information:**

The online version contains supplementary material available at 10.1186/s12992-021-00791-4.

## Introduction

The World Health Organization (WHO) recommends women should receive health interventions during routine maternity care visits (MCVs), including at least four antenatal care (4ANC) visits, institutional delivery assisted by skilled birth attendants (SBAs) [1], at least three PNC visits within the first week after childbirth [2]. These routine MCVs, from the conception to the first month of childbirth, is considered as maternal and newborn health (MNH) continuum of care (CoC) from the life cycle perspective [3]. The health of mothers and newborns is arguably a single entity except for their biological differences; interventions received by pregnant women can affect the health of newborns. Therefore, the MNH continuum is a vital period for the health of mothers and newborns; it is also a combined construct from survival and health service delivery [4]. A modelling study of 75 high-burdened countries estimated that increased coverage of essential MNH interventions could reduce up to 71% of neonatal deaths, 33% of stillbirths, and 54% of maternal deaths annually [5].

Globally, the MNH CoC has received substantial attention in research, policy, and programmes over the past two decades [4]. Sustainable Development Goal three (SDG-3) states universal coverage of quality MCVs across the CoC (target 3.8) [6, 7]. Out of nine tracer services in SDG-3, two are related to maternity and newborn care, such as childbirth assisted by SBAs and childbirth at health facilities (HFs) [8]. Thus, assessing a composite coverage of routine MCVs is important to track the utilisation status of tracer maternity health services and SDG-3 target 3.8.

However, the coverage of routine MCVs across the MNH CoC (i.e., during the antenatal through to postnatal period) is often low and characterised by high discontinuation rates at different stages of pathway. For example, the completion of all routine MCVs was low in several low- and middle-income countries (LMICs) [3, 4, 9, 10], including in Cambodia [9] and Tanzania [11]. For example, 90% dropout was reported in Tanzania from the first ANC visit to PNC visit, while the highest (55%) proportion was seen from institutional delivery to a PNC visit [11].

Nepal is one of the countries with the highest maternal mortality ratio (MMR) and neonatal mortality rate (NMR) in the South Asia region [12, 13]. Annually, 259 (per 100,000 live births) women die due to pregnancy and childbirth-related problems, and 21 (per 1,000 live births) newborns die within the first month of birth in Nepal [14]. High MMR and NMR may be contributed by low coverage of routine MCVs. For instance, the 2016 Nepal Demographic and Health Survey (NDHS) reported more than two-thirds of pregnant women received 4ANC visits, while nearly three in five women received institutional delivery and the first PNC visit within 48 h of childbirth [14]. Under the Aama and Newborn care programme in Nepal, 4ANC visits and institutional delivery are incentivized where women get some monetary incentives (e.g., women who complete at least four ANC visits in 4, 6, 8 and 9 months get 8 USD(1USD≈100 Nepali Rupees), and give birth at health facilities assisted by SBAs get an average of 20 USD) [15]. However, despite providing those maternity incentives, women with multiple forms of social disadvantages (e.g., women living in poverty, illiterate and disadvantaged ethnicities), and hard to reach-communities of remote provinces (e.g., Karnali, and Sudurpaschim) had the lowest coverage of ANC visits and institutional delivery compared to their privileged counterparts [16]. Such poor access to routine MCVs could result in a lack of utilisation of essential MNH interventions among populations with a high magnitude of MMR and NMR.

Other studies in Nepal have revealed that the utilisation of 4ANC visits contributed to the uptake of institutional delivery [17] and PNC visits [18], and women who utilised institutional delivery services were more likely to receive PNC visits [19]. In addition, a qualitative study reported that Nepalese women prioritise PNC visits if they experience any complications [20]. However, little evidence is available on the levels and social determinants associated with (dis)continuity of routine MCVs at the MNH CoC. Thus, this study aimed to examine the levels and social determinants of (dis)continuity of recommended MCVs across antenatal, delivery and postnatal period. Furthermore, findings of this study inform policymakers and programme managers to design and implement targeted policies to increase completion of all routine MCVs in Nepal. In addition, this study provides methodological insights how DHS data can be used to create composite coverage of all MCVs and measure the (dis)continuity care across the antenatal- postnatal pathway.

### Trajectory of Nepal’s health policy context for MNH

**Fig. 1:**
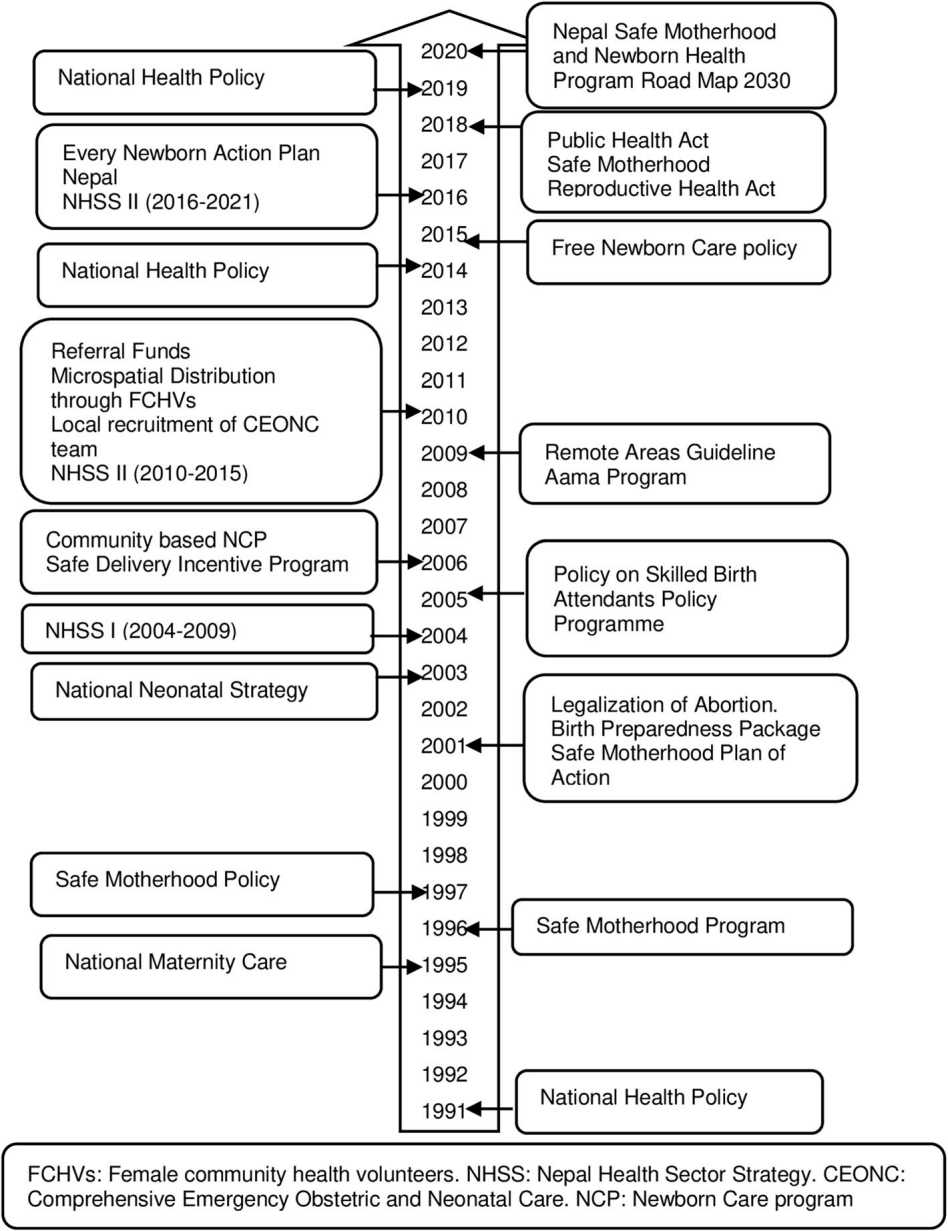
Timeline of selected policies, strategies, programmes, and plans for MNH in Nepal.

Figure 1 shows the major health policy, strategy, plans and programmes, particularly in MNH in Nepal over the last three decades[21]. National health policy 1991 increased the availability of health posts up to the village development committee (now called as wards). After the mid-2000s, Nepal has made significant policy shifts in maternal and newborn health, introducing a safe motherhood policy, safe delivery incentive programme, SBA policy and community-based newborn and child health programs. In the mid - 2010s, integration of maternal and newborn health services was given more attention, including free newborn care services. In addition, the quality of care is emphasised in the health policies and programmes such as Nepal Health Sector Strategy (2016-2021) and Nepal Safe Motherhood and Newborn Health Program Roadmap by 2030 [22].

## Methods

### Data source and sampling design

The NDHS 2016 data (https://dhsprogram.com/methodology/survey/survey-display-472.cfm) were used in this study [14]. The NDHS is a nationally representative cross-sectional survey conducted by Nepal’s Ministry of Health and Population (MOHP) in 2016 to identify primary health care coverage and health status, especially family planning, reproductive, newborn, child health, and nutrition. A more detailed sampling design is described in the 2016 NDHS report [14].In this study, we included 1978 women aged 15–49 years who had a live birth in the two years preceding the survey and provided information on their pregnancy, childbirth, and postnatal care [16].

### Country context- Nepal

Nepal is the landlocked country between China and India, with resident of 29 millions [23]. Nepal has diversities in languages and ethnicities. Brahmin and Chhetri are the dominant ethnicities, and Nepali is the national language and is primarily spoken in the Hilly areas [24]. In 2015, passing with different political turmoils, Nepal reformed 240 years long unitary monarchial political system to a federal republic country. In line with the federal governance system, the health system is also decentralized into three layers of governments: one federal, seven provincial, and 753 local governments [25]. Municipalities are further divided into several wards (minimum 5 to maximum 35 wards) for administrative and service delivery. Nepal has a mixed care delivery system, and health services are provided through public and private providers/hospitals. Basic health services are provided from public facilties through public fundings, while people have to pay for secondary and tertiary health services. These health services in public facilities are relatively cheaper than private facilities; however, there is still high out-of-pocket expenditure (57% of current health expenditure) [26]. Nepal has a national health insurance scheme, a voluntary health insurance scheme implemented in 2016 [27]; however, enrolment is low, and there is a high dropout rate for the renewal of the insurance programme.

### Conceptual framework

**Fig. 2:**
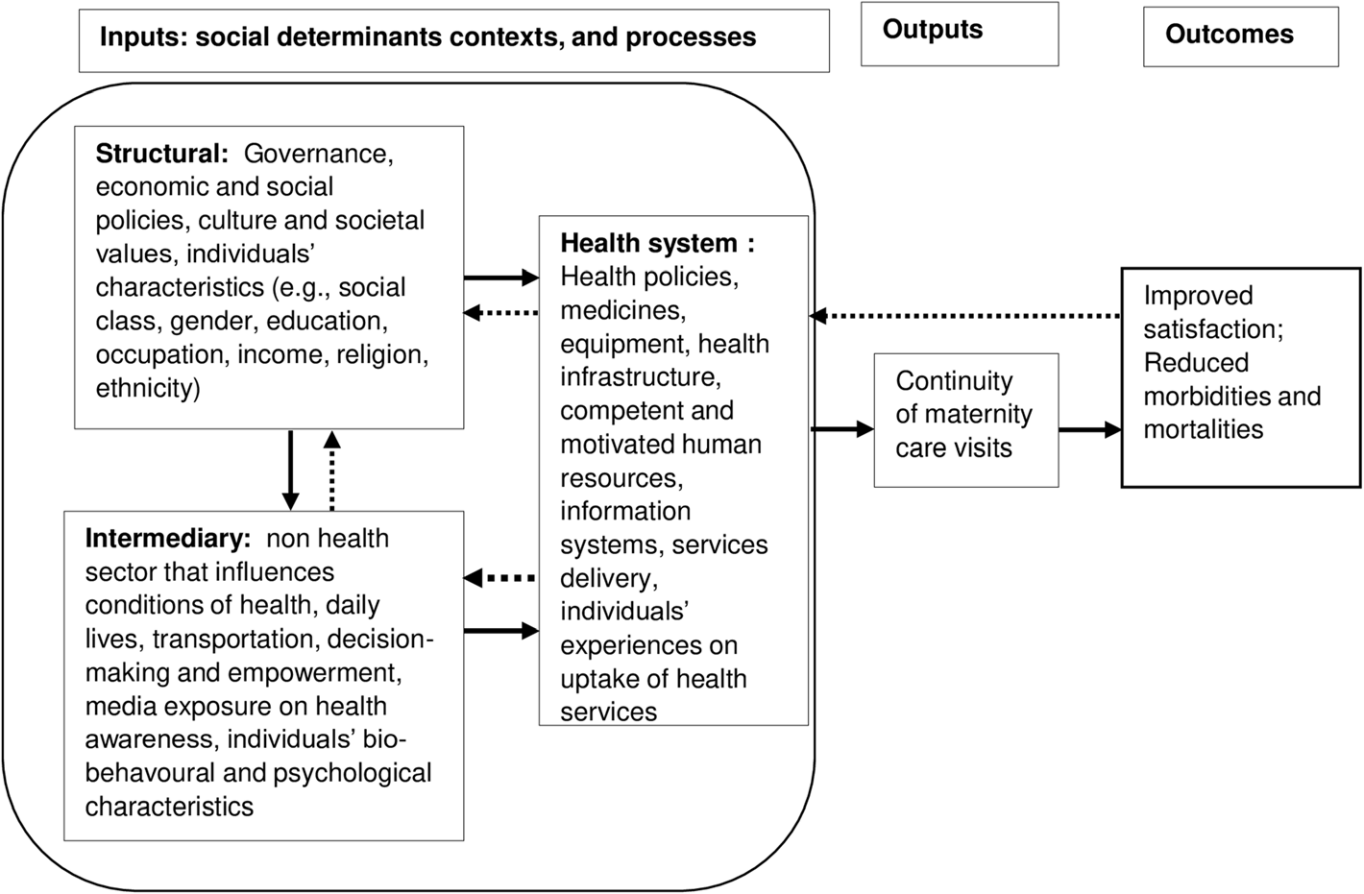
Conceptual framework to guide the study.

Based on the previous conceptual framework [28, 29], we developed a conceptual framework [30] and adapted it for this study to guide the analysis and interpretation (Fig. 2). The conceptual framework comprises the input-output-outcome model. We grouped several social determinants as inputs into three broader domains structural, intermediary, and health system. Structural social determinants are included to identify the most disadvantaged women groups (who are left behind). Similarly, intermediary social determinants influence the conditions of health, cover non-health sector factors, and can be addressed through multisectoral actions. Finally, health system factors are concerned with health care delivery.

### Study variables

The detail of independent variables, including women’s socioeconomic characteristics and health-seeking factors, are described in the Supplementary file (Table S1). Briefly, structural social determinants included women’s ethnicity, wealth status, education, religion, maternal occupation, perceived violence, decision-making for at least three areas (healthcare, purchasing, and movement), and sex of the household head. Intermediary variables were women’s language, maternal age, residence, provinces, region, birth order, sex of child, access to bank account, media exposure, perceived problem of distance to HFs, and intended birth of the last child. Finally, the health system variables included the women’s (perceived) problem of not having female health workers, awareness on health mothers’ groups in the community, and mode of delivery (normal or caesarian section). We further categorised some socioeconomic variables such as ethnicity (advantaged and disadvantaged), education (illiterate, primary, secondary and higher), wealth status (upper and lower) [16].

This study used the data from the 2016 NDHS, and where responses were self-reported based on the recall of women who had a live birth two years before the survey. Along the pathway of the antenatal-postnatal period, there were three possible points of discontinuation: before completing 4ANC visits, completion of 4ANC visits but discontinued institutional delivery, completion of 4ANC visits and institutional delivery but discontinued PNC visit. So, in this study, the outcome variable was created using the information on the utilisation of maternity service in the antenatal, delivery and postnatal period. The outcome variable had four mutually exclusive categories: had no or less than 4ANC visits=1; had 4ANC visits but no institutional delivery (ID) =2; had 4ANC visits and ID but no PNC (mother-newborn pair) visit=3; had 4ANC visits and ID and PNC visit=0 (reference category).

### Data analysis

Multinomial logistic regression analysis was conducted, and the magnitude of (dis)continuity of care was reported as relative risk ratios (RR) with 95% confidence intervals (CIs). Sampling weights (available in the NDHS 2016 dataset) have been applied in the analysis, so results are representative at the national and strata levels. All analyses were weighted to adjust for the two-staged cluster sampling used in the 2016 NDHS [14]. All estimates were reported in weighted value (unless otherwise indicated) including frequency, and proportion (%). The clustering effect of complex sampling design was adjusted using survey ‘svy’ set command in Stata 14.0 (Stata Corp, 2015).

Before running the multivariable multinomial regression model, multicollinearity was checked and excluded independent variables having variation inflation factors ≥3 [31]. Backwards elimination multivariable multinomial logistic regression analyses were conducted [32]. First, the full multinomial multivariable regression model was run, estimated p-value for each independent variable. Then, the most insignificant variable (variable with the highest p-value) was deleted, comparing p values with other independent variables. This procedure was repeated until no insignificant independent variable was left at *p*<0.20 [33]. The statistical significance level was set *p*<0.05 (two-tailed) to identify the independent variables associated with the outcome variable. The goodness of fit test was conducted using the Log-likelihood Ratio test [6].

## Results

### Background characteristics of women

The background characteristics of women included in this study are presented in supplementary file 1, Table S2. In summary, among the 1,978 women, 42% were from households in the lower wealth status, while more than two-thirds (69%) of women were from disadvantaged ethnicities. Nearly two in five women (42%) were native Nepali speakers. More than one in four women (26%) were from province two, only 6% were from Karnali province. Similarly, more than half (55%) of women were from the Terai (Plain) Region, but about half (46%) of women were from urban areas. Two-thirds (67%) of women had no decision-making authority to health-seeking, buying something and nearly one-third (29%) of women reported any kind of perceived violence (see variable detail description in Supplementary file 1, Table S1). Four in five (79.7%) women were aged 20–34 years, and approximately 69% did not have a bank account. Over two-thirds (68%) of women had no awareness of the availability of a health mothers’ group in their communities. Three in five (60%) women felt distance to an HF was a challenge when accessing health services. More than two thirds (72%) of women perceived it as challenging to access care when there were no available female health workers (Supplementary file, Table S2).

### (Dis)continuity of care of routine MCVs at different stages of CoC

**Fig. 3:**
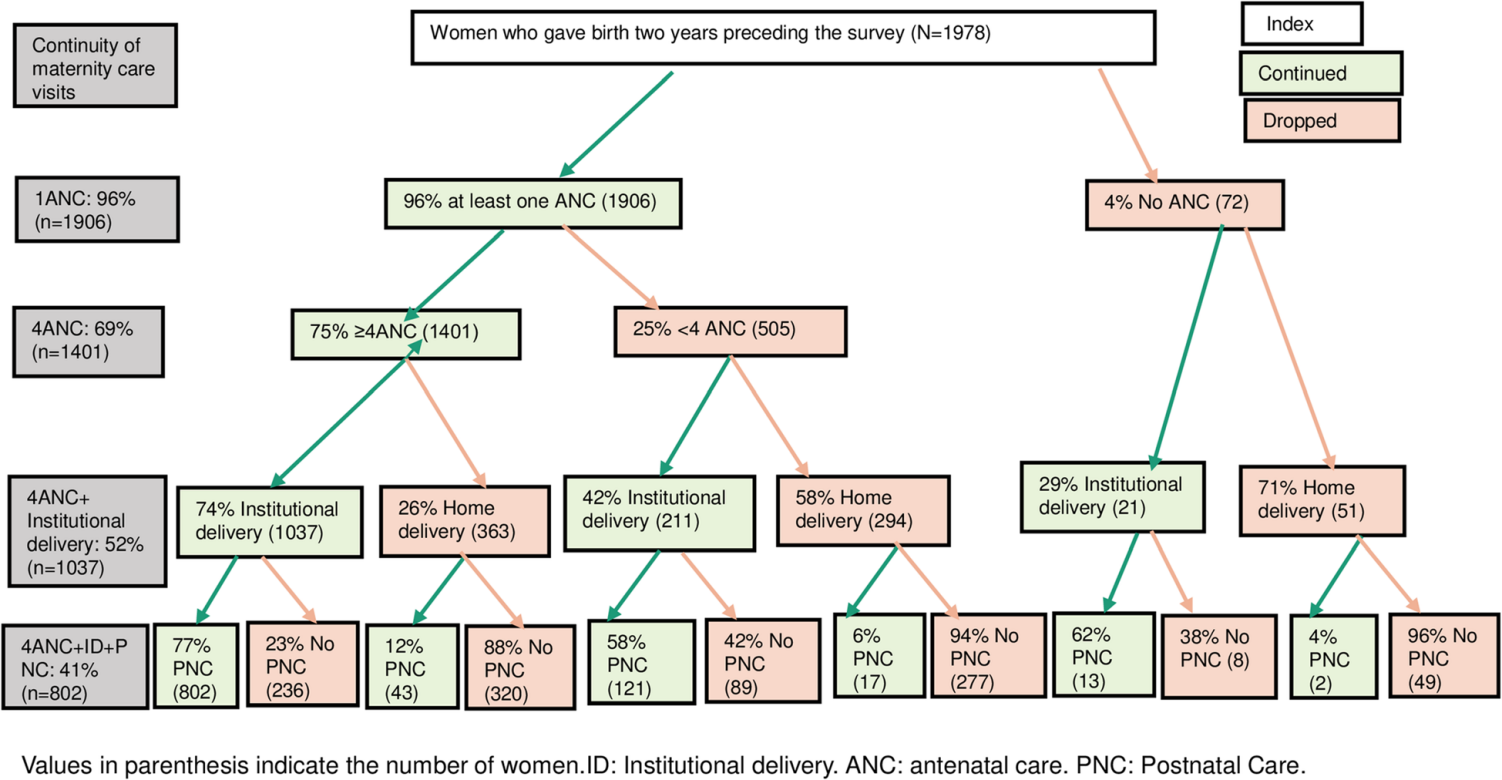
(Dis)continuity of routine MCVs across the antenatal-postnatal period in Nepal.

Figure 3 shows the continuity of routine MCVs across the MNH continuum of care. Among 1,978 women included in this analysis, only two in five (41%) attended all three MCVs (4ANC visits, institutional delivery, and one PNC visit within 48 h of childbirth). Almost all (96%) received at least one ANC visit, but only 71% completed 4ANC visits. More than one in two women (52%) completed at least 4ANC visits and received institutional delivery services. Women without 4ANC visits, however, had a higher home delivery rate. For instance, among women who were unable to complete 4ANC visits, 58% of them gave birth at home, while 71% of women with no ANC visits (*n*=72) were delivered at home. Only 4% (of *N*=1,978) of women did not receive ANC visits, institutional delivery, or PNC visits (Fig. 3).

**Fig. 4:**
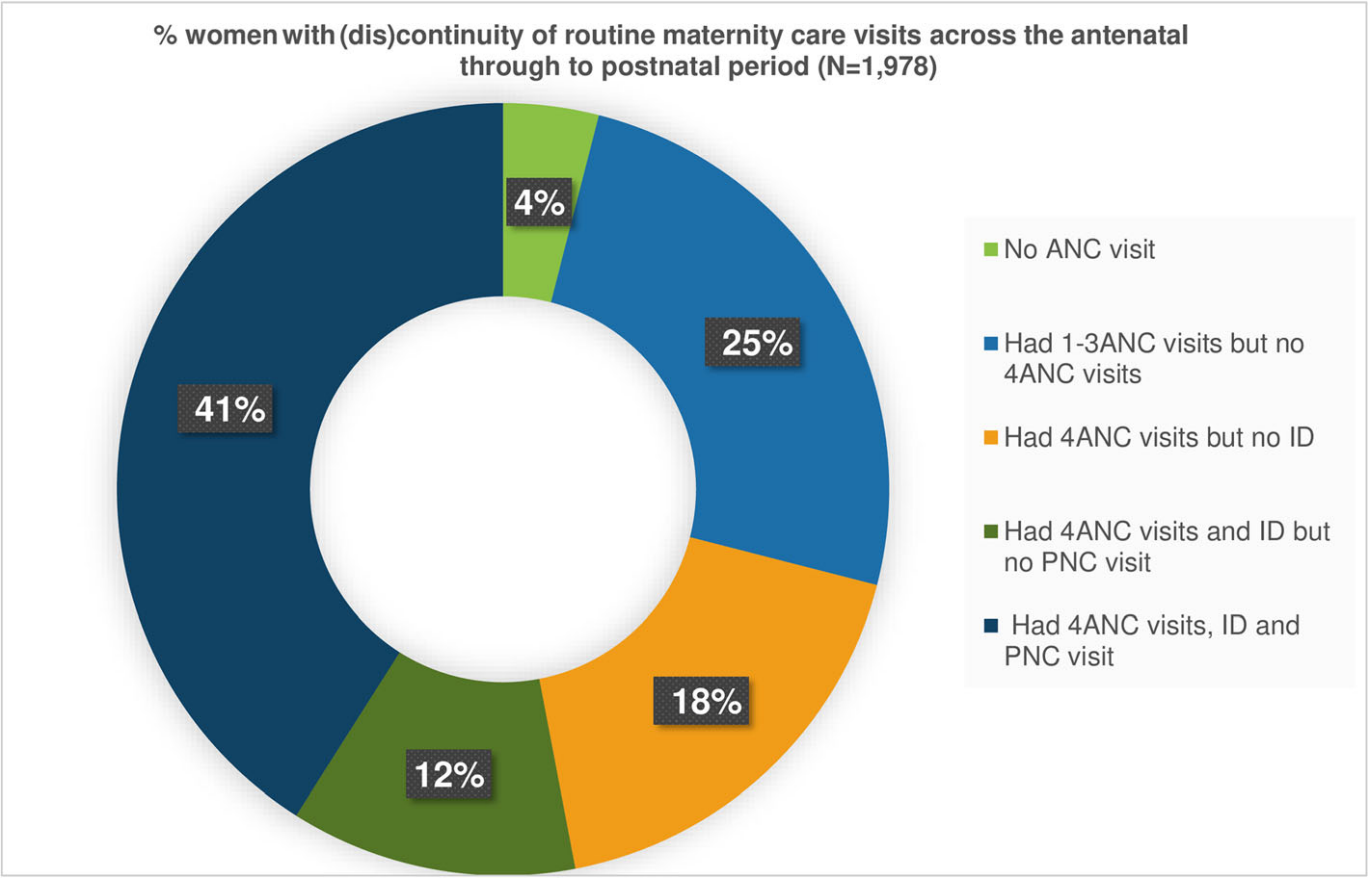
Discontinuity of care along the pathway of antenatal through to the postnatal period.

Additionally, Fig. 4 shows the proportion of women who discontinued maternity care along the antenatal-postnatal pathway. Nearly one in 20 women did not visit any forms of routine maternity care visits. About one in four (25%) women had at least one ANC visit but not completed, while one in five women had at least 4ANC visits but did not complete institutional delivery. Only two in five women completed all recommended 4ANC visits.

### Descriptive analysis of (dis)continuity of care of MCVs

Table 1 shows the women who completed/discontinued routine MCVs across the maternal and newborn health continuum. Over half of women completed all visits if they were belonged to advantaged ethnicity (54%), had secondary or higher-level education (54%), had jobs (53%), had a bank account (54%). The completion of all MCVs was higher if women were from provinces three (51%) and four (54%). Similarly, their completion of MCVs if women had media exposure (51%), distance to HF was not a problem (53%), and who delivered via C-Sect. (71%). However, only one in four women completed all three routine care visits if they were from Karnali province (24%), a Bhojpuri speaker (21%), illiterate (25%), and higher birth order (≥4) (21%) (Table 1).

**Table 1:** (Dis)continuity of routine MCVs during maternal and newborn health continuum of care in Nepal, 2016 (*N*=1,978)

social determinants	Frequency	Had less than 4ANC visits (%)	Had 4ANC visits but no ID (%)	Had 4ANC visits and ID but no PNC visit (%)	Had 4ANC visits ID and PNC visit (%)	p
**Structural**						
**Wealth status**						
Lower (40%)	832	35.1	26.2	8.6	30.0	<0.001
Upper (60%)	1,146	24.8	12.7	14.3	48.2	
**Ethnicity**						
Disadvantaged	1,374	34.3	19.6	11.3	34.8	<0.001
Advantaged	604	17.6	15.5	13.4	53.6	
**Religion**						
Others	306	39.1	16.2	10.1	34.5	0.056
Hindu	1672	27.3	18.8	12.2	41.7	
**Maternal education**						
Illiterate	570	46.5	20.2	8.8	24.6	<0.001
Primary	391	36.5	24.9	10.0	28.6	
Secondary or more	1,016	16.6	14.8	14.4	54.1	
**Maternal occupation**						
Not working	928	32.9	13.7	12.9	40.5	<0.001
Agriculture	823	27.6	25.0	10.0	37.3	
Working paid	227	19.2	13.6	14.5	52.7	
**Perceived violence**						
No	1,397	27.8	18.1	11.2	42.9	0.044
Yes	581	32.4	19.0	13.7	34.9	
**Decision-making**						
No	1,324	30.9	18.9	11.6	38.6	0.111
Yes	654	25.6	17.3	12.6	44.5	
**Household head**						
Male	1,438	30.4	18.9	12.2	38.6	0.073
Female	540	25.9	17.0	11.2	45.9	
**Intermediary**						
**Language**						
Nepali	839	19.8	17.8	13.2	49.2	<0.001
Maithili	360	32.4	24.5	11.2	32.0	
Bhojpuri	267	54.4	12.1	12.2	21.3	
Others	512	29.1	18.2	10.1	42.6	
**Residence**						
Urban	1,062	24.5	14.0	13.5	48.0	<0.001
Rural	916	34.6	23.4	10.0	32.0	
**Provinces**						
One	338	21.2	23.0	8.3	47.5	<0.001
Two	513	42.0	19.2	12.0	26.9	
Bagmati	312	24.2	14.2	10.7	50.9	
Gandaki	164	24.7	11.9	9.0	54.4	
Lumbini	364	25.3	19.5	13.5	41.7	
Karnali	121	45.4	22.0	9.0	23.6	
Sudurpaschim	166	16.0	15.5	23.0	45.5	
**Region**						
Mountain	131	28.3	30.5	3.4	37.8	0.001
Hills	760	23.8	18.2	11.7	46.3	
Terai	1,087	33.0	17.0	13.1	36.9	
**Maternal age (years)**						
<19	291	25.8	15.7	16.9	41.6	0.146
20-34	1,582	29.2	18.8	11.1	40.9	
≥35	106	37.7	19.7	10.2	32.4	
**Birth order**						
<4	1,678	24.9	17.7	13.3	44.1	<0.001
≥4	300	52.8	22.3	4.4	20.6	
**Sex of index child**						
Male	1,063	28.9	17.4	12.1	41.6	0.699
Female	915	29.5	19.5	11.7	39.4	
**Access to bank account**						
No	1,367	33.9	20.2	11.6	34.4	<0.001
Yes	611	18.6	14.3	12.7	54.4	
**Media exposure**						
No	911	40.0	22.6	9.5	27.8	<0.001
Yes	1,067	19.9	14.7	13.9	51.4	
**Last child (index child)**						
Unwanted	418	39.7	16.4	11.1	32.9	<0.001
Wanted	1,560	26.3	18.9	12.1	42.6	
**Distance to HFs as a perceived problem**						
No problem	763	23.0	13.6	12.2	51.3	<0.001
Big problem	1,215	33.0	21.4	11.8	33.8	
**Health system**						
**Perceived problem not having female providers**						
No problem	562	22.1	14.1	10.7	53.1	<0.001
Big Problem	1,416	31.9	20.1	12.4	35.6	
**Awareness of health mothers’ group**						
No	1,340	32.5	17.7	11.5	38.3	<0.001
Yes	638	22.1	19.8	12.8	45.4	
**Mode of delivery**						
Normal	1,780	30.6	20.4	11.8	37.2	<0.001
C-section	198	16.1	0	13.0	70.9	

ID: institutional delivery, HF: health facility, HMG: health mothers’ group, p-values obtained from Fisher exact test. Other languages include (e.g., Tharu, Magar). Other religions include Buddha, Jain, Kirat, Christian.

### Correlates of the (dis)continuity of MCVs across the maternal and newborn health continuum

Findings of the bivariable regression analysis are presented in Supplementary file (Table S3). Correlates of discontinuation of MCVs in the bivariable analysis were structural (wealth status, education), intermediary (residence, province, region, birth order, media exposure on public health issues, access to a bank account, the intention of last birth, perceived problem of the long distance to the HFs, and perceived violence) and health system (perceived problem if not having female providers in HFs, awareness of health mothers’ groups, and mode of delivery).

Table 2 shows the multivariate multinomial regression analysis of correlates of discontinuity of MCVs during the antenatal-postnatal period. Eight determinants were significantly associated with discontinuity of care before completing 4ANC visits. For example, there was a higher relative risk of discontinuation before completion of 4ANC visits (completing all MCVs) if women were illiterate (Relative Risk Ratio (RR)=2.65; 95% CI: 1.72, 4.08), from lower wealth status (RR=2.39; 95% CI: 1.63, 3.51), speak Bhojpuri language (RR=3.28; 95% CI: 1.26, 8.58), or live in Karnali province (RR=4.08; 95% CI: 2.30, 7.21). Similarly, if women had high birth order (≥4) (RR=2.15; 95% CI: 1.41, 3.30), women not having media exposure (RR=1.81; 95% CI: 1.33, 2.46), unwanted last birth (RR=2.11; 95% CI: 1.47, 3.02), who were not aware of health mothers’ group in their community (RR=1.53; 95% CI: 1.13, 2.07) had a higher risk of discontinuing before completing 4ANC visits.

Similarly, nine determinants were significantly associated with continuity of care until 4ANC visits but discontinued before completing institutional delivery (Table 2). Women completed 4ANC visits but discontinued institutional delivery(compared to the completion of all three MCVs) if they had primary education (RR=1.92; 95% CI:1.26, 2.93) and lower wealth status (RR=2.82; 95% CI:1.88, 4.22), who were involved in agriculture (RR=1.51; 95% CI:1.04, 2.19) or from disadvantaged ethnicity (RR=1.54; 95% CI:1.05, 2.26) compared to women with higher education, who belonged to upper wealth status, who were housewives, and advantaged ethnicities, respectively (Table 2). Moreover, women who lived in rural areas (RR=1.91; 95% CI: 1.36, 2.69) and women with no media exposure (e.g., radio, newspaper, television) (RR=1.56; 95% CI: 1.13, 2.14) had a higher risk of discontinuity of care before completing institutional delivery compared to women from urban areas.

Moreover, two determinants were associated with continuity of care until 4ANC visits and institutional delivery but discontinued before completing the PNC visit (Table 2). For instance, women from provinces Karnali (RR=2.24; 95% CI: 1.07, 4.71) and Sudurpaschim (RR=3.57; 95% CI: 1.87, 6.81), and women with a perceived problem of not having a female provider (RR=1.64; 95% CI: 1.12, 2.39) had a higher risk of completing all 4ANC visits and institutional delivery but discontinued before completing PNC visit.

**Table 2:** Correlates of (dis)continuity of routine MCVs across the antenatal through to postnatal period in Nepal, NDHS 2016 (*N*=1,978)

Social determinants	Had no or less than 4ANC visits (ARR; 95% CI)	Had 4ANC visits but no ID (ARR; 95% CI)	Had 4ANC visits and ID but no PNC visit (ARR;95% CI)
**Structural**			
**Wealth status**			
Upper (60%)	1.00	1.00	1.00
Lower (40%)	2.39 (1.63, 3.51) ***	2.82 (1.88, 4.22) ***	0.93 (0.59, 1.48)
**Ethnicity**			
Advantaged	1.00	1.00	1.00
Disadvantaged	1.47 (0.79, 2.71)	1.54 (1.05, 2.26) *	1.28 (0.82, 1.98)
**Maternal occupation**			
Agriculture	0.79 (0.56, 1.11)	1.51 (1.04, 2.19) *	0.87 (0.59, 1.29)
Housewife	1.00	1.00	1.00
Working paid	0.64 (0.35, 1.15)	1.22 (0.70, 2.13)	1.08 (0.62, 1.90)
**Maternal education**			
Higher	1.00	1.00	1.00
Illiterate	2.65 (1.72, 4.08) ***	1.39 (0.93, 2.08)	1.21 (0.72, 2.02)
Primary	2.41 (1.62, 3.57) ***	1.92 (1.26, 2.93) **	1.20 (0.74, 1.93)
**Intermediary**			
**Language**			
Nepali	1.00	1.00	1.00
Maithili	1.31 (0.57, 3.00)	1.36 (0.6, 3.11)	0.71 (0.29, 1.77)
Bhojpuri	3.28 (1.26, 8.58) *	0.97 (0.35, 2.68)	1.06 (0.44, 2.57)
Others	1.52 (0.81, 2.84)	0.89 (0.55, 1.42)	0.66 (0.41, 1.05)
**Province**			
One	1.00	1.00	1.00
Two	1.73 (0.93, 3.21)	1.34 (0.67, 2.71)	2.19 (0.89, 5.42)
Bagmati	1.31 (0.68, 2.54)	0.75 (0.41, 1.35)	1.14 (0.57, 2.27)
Gandaki	1.45 (0.79, 2.68)	0.47 (0.23, 0.94) *	0.95 (0.46, 1.97)
Lumbini	1.10 (0.61, 1.96)	1.09 (0.67, 1.76)	1.93 (0.98, 3.82)
Karnali	4.08 (2.30, 7.21) ***	1.32 (0.65, 2.68)	2.24 (1.07, 4.71) *
Sudurpaschim	0.56 (0.29, 1.07)	0.51 (0.25, 1.02)	3.57 (1.87, 6.81) ***
**Residence**			
Urban	1.00	1.00	1.00
Rural	1.35 (0.95, 1.93)	1.91 (1.36, 2.69) ***	0.97 (0.64, 1.48)
**Maternal age (in years)**			
15-19	0.79 (0.52, 1.20)	0.63 (0.39, 0.99) *	1.24 (0.78, 1.95)
20-34	1.00	1.00	1.00
35 or above	0.55 (0.29, 1.04)	0.79 (0.35, 1.78)	1.66 (0.67, 4.11)
**Birth order**			
<4	1.00	1.00	1.00
≥4	2.15 (1.41, 3.30) ***	1.5 (0.98, 2.30)	0.52 (0.26, 1.06)
**Media exposure**			
Yes	1.00	1.00	1.00
No	1.81 (1.33, 2.46) ***	1.56 (1.13, 2.14) **	1.01 (0.69, 1.50)
**Last birth (index child)**			
Wanted	1.00	1.00	1.00
Unwanted	2.11 (1.47, 3.02) ***	1.15 (0.76, 1.72)	1.10 (0.67, 1.82)
**Health system**			
**Perceived problem not having female providers**			
No problem	1.00	1.00	1.00
Big problem	1.25 (0.89, 1.76)	1.46 (1.05, 2.04) *	1.64 (1.12, 2.39) *
**Awareness on health mothers’ group**			
Yes	1.00	1.00	1.00
No	1.53 (1.13, 2.07) **	1.10 (0.79, 1.53)	1.06 (0.76, 1.46)

*** *p*<0.001, ** *p*<0.01, * *p*<0.05. Results obtained from the multinomial logistic regression analysis. Determinants that had *p*<0.2 included in the final model adjusting for covariates listed in the table. The likelihood ratio of the reduced model with the full model was [chi-square=19.47; *p*=0. 0.555], and our model was the best fit. HWs: health workers, HMG: health mothers’ group, HF: health facility, ID: institutional delivery, ANC: antenatal care. The reference category of outcome variable was the completion of all three MCVs. Other languages include Tharu, Magar. ARR: Adjusted risk ratio.

## Discussion

This study examined the composite coverage of routine MCVs. Only 41% of women received all three routine MCVs, higher discontinuation from antenatal through to postnatal period. There was a high proportion of discontinuation around later gestational weeks of pregnancy (4ANC visits) and institutional delivery. Several correlates were associated with the (dis)continuity of MCVs across the MNH continuum. For instance, women with structural disadvantages (e.g., disadvantaged ethnicity, lower wealth status, illiterate women) had a higher risk of discontinuing routine MCVs. Higher discontinuity of MCVs was reported if they were from Karnali and Sudurpaschim provinces, who speak Bhojpuri, had high birth order, and poor media exposure on health issues, who had poor awareness on the health mothers’ group, and who perceived problems if not having female providers.

The reasons for low completion of routine MCVs may be due to high discontinuation at later gestational weeks of pregnancy or around childbirth. This study identified that women with home delivery were more likely to dropout the first PNC visit. Past studies in Nepal have reported poor uptake of 4ANC visits and institutional delivery due to long walking hours, and unavailability of infrastructure and equipment for childbirth services in local health facilities [34, 35]. The current study revealed lower discontinuation from institutional delivery to PNC visits than previous antenatal and intrapartum visits. The reasons for this could be due to the compulsory PNC checkup at health facilities before discharge in case of facility birth provisioned in the National Safe Motherhood Programme [36]. However, evidence revealed low PNC visit than institutional delivery[16], which suggest not all women who gave birth at health facilities were not getting PNC services before they discharge from health facilities. The lower continuity of recommended MCVs was consistent with the studies in Cambodia [37] and Lao PDR [38]. In later weeks before childbirth or during childbirth, pregnant women may face difficulties reaching health facilities if physical access is poor or there is no suitable accommodation close to the health facility in Nepal [19]. Even where there was good accessibility of health facilities, knowledge of and demand for PNC was low in Nepal [39]. The upgrading and accrediting of all health posts to birthing centers and strengthening existing birthing centers could increase the availability of quality intrapartum care in rural Nepal. On the other hand, ensuring necessary arrangements at health facilities (e.g., providing medicine and equipment and training female community health workers), including transportation facilities to reach health facilities, could increase institutional delivery and PNC visit.

The current study revealed women with social disadvantages (e.g., illiterate, poor, marginalised ethnic group, involved in agricultural work) and geographical factors had higher discontinuation across the MNH continuum. In Nepal, generally, women with social disadvantages have difficulties in daily living and working conditions, usually have more focus on livelihood support than healthcare. They have inequitable distribution of livelihood opportunities and resources that contribute to poor access to and higher discontinuation of MCVs[19]. Earlier studies in Nepal also have reported women’s living, and working life in the mountains [35] and women with poor wealth status also hindered the utilisation of MCVs services [40]. Such women in the maternity and postnatal period may not seek health services unless there are complications [20]. These factors are mostly non-modifiable and often require long term sociopolitical interventions [29, 41–43], and technical and biomedical focussed approaches on their own may not improve routine MCVs across the maternity continuum [42]. In the short term, Nepal’s Ministry of Health and Population can focus its programmes on targeting women living in difficult geographical areas (e.g., Karnali province) and women with social disadvantages (e.g., poor, marginalised ethnicity). A previous study found to improving health services delivery included strengthening birthing centers (e.g., health logistics, human resources and training), and establishing maternity waiting homes [44]. Childbirth services in all rural health facilities could increase the institutional delivery assisted by SBAs and first PNC visit. The PNC home visit and counselling on maternity and newborn care services through female community health volunteers (FCHVs) in hard-to-reach communities of remote provinces (e.g., Karnali) could also improve PNC services. Longer-term structural interventions to improve the uptake of routine MCVs may include improving female access to formal and informal education and employment opportunities [29].

The study found higher discontinuation of MCVs across the MNH CoC, especially women who had more than four children, or women who had last birth unintended. However, health system strategies could improve the continuity of MCVs across the CoC, such as birth spacing, awareness of health issues through mass media exposure, and having female providers at the health facilities. This suggests if women had intended pregnancy, they could prioritise routine MCVs for healthy pregnancy and childbirth. In addition, effective uptake of family planning (FP) services could help for wanted pregnancy and reduced birth spacing, resulting in women being more likely to complete recommended routine MCVs for their intended birth [45].

Health awareness on the importance of pregnancy, childbirth, and PNC services can be improved via exposure to mass media (e.g., local radios, newspaper, television) and dissemination of health information to current and future mothers. A past study in Nepal reported that mass media exposure was positively associated with maternal services [45]. Other studies in other LMICs settings showed that health awareness through digital tools such as mobile phones could play an important role in utilising health services [46] and maternity services [47]. In the current digital era, the use of these digital tools and social media platforms can be helpful to raise awareness on maternity and newborn care and information on health services. In addition, context-specific strategies can be adopted to increase the uptake of needed MCVs that include outreach clinics in remote and underprivileged communities or mobilisation of local community workers for PNC home visits [48]. The health mothers’ groups can raise awareness in the community; however, most of these groups are not functional regularly in Nepal [49]. Health mothers’ groups are FCHVs-led community health groups where current and future mothers can gather and discuss reproductive, maternal, child health and nutrition issues [50]. Thus, properly functional health mothers’ group in the community could raise awareness among pregnant women and provide necessary health information in their pregnancy and childbirth. Such health groups could address the social taboos as talking about reproductive health-related issues is culturally taboo in Nepali society, and women are usually like to share the provision of female providers [20, 51]. Thus, such awareness raising programmes need to focus to disadvantaged population groups (e.g., women of lower wealth status, who speak Maithili language, living in remote areas). The Aama programme (maternity incentive programme) has provisioned 4ANC visits and institutional delivery, resulted in the increased coverage of these visits; however, there is no provision of financial incentive for PNC visits [52]. In the current Aama programme, in addition to 4ANC visits and institutional delivery, incentivizing and integrating PNC visits could potentially increase the completion of routine visits in the maternity care continuum. The health promotion activities such as health education and advocacy, maternal newborn and child health services and context-specific policy need to be ensured. Such programs can be targeted to marginalised women such as Dalits, Karnali province, and Bhojpuri speaking women.

The measurement of continuity of care is vital for tracking health services coverage across the MNH continuum by creating a composite coverage indicator of all three routine MCVs. Such measurement and tracking could give the actual coverage of MCVs across the CoC. Past studies reported routine monitoring systems [15] lack composite coverage of all routine MCVs across the CoC [53, 54]. Completing all routine maternity care visits is a critical window of opportunity to receive recommended MNH interventions for the survival of mothers and newborns. A modelling study estimated that increased access and quality of MNH interventions across the CoC could avert up to two-thirds of maternal deaths, more than half of maternal deaths, and nearly one-third of stillbirths annually [5]. The composite coverage measurement of maternity MCVs can have a significant implication to track the coverage in countries with high maternal and neonatal deaths. The composite indicator can be created and included in routine health management information systems (HMIS) and periodic health surveys (e.g., demographic and health surveys). In addition, the composite coverage indicator can help to track services coverage among disadvantaged populations and can be employed to assess the reach of existing policy interventions. The monitoring of routine quality maternal and newborn health services is prioritised in SDG-3 [55], the creation and integration of composite coverage indicator can be the initial step to achieve this milestone.

### Implications for policies and programmes

The composite coverage indicators of the maternity care continuum can have implications in tracking services utilisation among disadvantaged populations. The findings of this study can have potential implications in the revision of existing maternity incentive programmes. This study created and executed two composite coverage indicators across the maternity care continuum (i) combined coverage of 4ANC visits and institutional delivery indicator can be used to revise the existing Aama program (maternity incentive programme), and (ii) combined coverage of all three MCVs can be employed to extend the scope of it incorporating PNC visit. First, the current Aama program has provided the incentive for 4ANC visits and institutional delivery separately; instead, these two can be merged and targeted to those who complete 4ANC visits and institutional delivery. Second, the composite coverage of recommended MCVs can be used to extend the maternity incentive program by incorporating the PNC visit across the antenatal, institutional delivery and postnatal period. In addition, the current Aama program has provisioned maternity incentives to all women irrespective of marginalisation status. But such incentives need to be customized to women with multiple forms of marginalisation in the context of the beneficiaries from corresponding local government and wards. Nepal has implemented a decentralised health system, and local health officers can create composite coverage indicators and track the composite coverage of MCVs in their catchment areas. Municipal governments are close to communities; they can identify the most disadvantaged population groups and track the composite coverage of MCVs. Such measurement and tracking services coverage among those groups might help provide financial incentives for completing all recommended visits.

## Strengths and limitations

This study has a few strengths and limitations. Strengths included; first, this study is based on a nationally representative survey with a high response rate (98%), and findings could be generalised at the national level. Second, this study considered the PNC visit for mothers and newborns rather than previous studies that examined PNC visit for newborn or PNC mothers separately. Thirdly, creating composite indicators of all three MCVs and analysis can provide a new perspective. Finally, other researchers can adopt similar analyses using multiple rounds of DHS surveys. This study has the following limitations. First, as this study used the 2016 NDHS data based on the observational and cross-sectional design, inferences drawn from this study allow the study of correlations rather than causality. Second, the NDHS 2016 collected information based on recall of women who had a live birth five years before the survey (2011-2016); however, this study included a short recall period of two years, restricting the study sample (2014–2016). Third, this study is based on secondary data analysis; we could not include important variables, including obstetric complications that could contribute to discontinuation along the pathway. Fourth, the outcome variable was self-reported after face-to-face interviews with women, which may have social desirability bias. Finally, this study has not explored stories of why women discontinued health services utilisation across the CoC. The qualitative research could provide a deeper understanding of real stories of the underlying reasons for discontinuation across the CoC.

## Conclusions

Women had high proportion of discontinuation around the late gestational week of childbirth. Disadvantaged women had high discontinuation in different stages of the maternity care continuum. Creation and execution of composite coverage of 4ANC visits, institutional delivery and PNC visit could track the uptake of health services across the CoC. Monitoring MCVs utilisation using composite coverage indicators and provision of focused strategies (e.g., home visits and outreach services, incentives who complete all routine MCVs) could increase the completion of all routine MCVs across the CoC, especially among disadvantaged women. The continuous availability of maternity and newborn services in health facilities and trained female health providers could improve the continuity of maternity care during pregnancy, intrapartum, and the postnatal period. In the decentralised health system of Nepal, the local rural municipality can be responsible for funding to design and execute these strategies for universal coverage of completion of all routine MCVs and better maternal and neonatal outcomes.

## Supplementary information

Additional file 1 **Table S1.** Description of variables included in the analysis of (dis)continuity of routine MCVs in Nepal, NDHS 2016. **Table S2.** Characteristics of women who had a live birth in the two years preceding the survey in Nepal in NDHS 2016. **Table S3.** Bivariable multinomial logistic regression analysis of (dis)continuity of routine MCVs in Nepal, NDHS 2016.

## Data Availability

Data used in this study are publicly available secondary data obtained from the DHS **(**https://dhsprogram.com/data/available-datasets.cfm) .
