# Supplementary material for: Universal coverage of the first antenatal care visit but poor continuity of care across the maternal and newborn health continuum among Nepalese women: analysis of levels and correlates
Source: Global Health. 2021 Dec 11;17:141. doi: 10.1186/s12992-021-00791-4 (PMC8665493; doi:10.1186/s12992-021-00791-4)
Supplement: Supplementary file 1 — Table S1. Description of variables included in the analysis of (dis)continuity of routine MCVs in Nepal, NDHS 2016. Table S2. Characteristics of women who had a live birth in the two years preceding the survey in Nepal in NDHS 2016. Table S3. Bivariable multinomial logistic regression analysis of (dis)continuity of routine MCVs in Nepal, NDHS 2016. [file 12992_2021_791_MOESM1_ESM.doc]

## Supplementary file

Table S1: Description of variables included in the analysis of (dis)continuity of routine MCVs across the MNH continuum in Nepal, NDHS 2016

| **Correlates** | **Categories** | **Descriptions** |
| --- | --- | --- |
| **Independent variables** | | |
| **Structural** |  |  |
| Wealth status | Lower wealth status (lower 40%); Upper wealth status (upper 60%) | NDHS data had a variable wealth quintile, which was calculated based on scores generated from principal components analysis of households' assets (40 items). It was grouped into two categories: lower (poorest, poor, or collectively called as lower two quintiles); and upper (middle, richer, and richest or collectively called as upper three quintiles) wealth status. |
| Ethnicity | Disadvantaged; Advantaged | Disadvantaged: Dalit, Muslims, and Terai caste, Janajatis disadvantaged) and advantaged: Brahmin/Chhetri, advantaged Janajatis). |
| Maternal education | Illiterate; Primary; Secondary or higher | Illiterate: Cannot read and write; Primary: 1-8 grade.  Secondary or higher: 9^th^ grade and higher |
| Religion | Others; Hindu | Others include Muslims, Jain, Christian, Buddhist |
| Maternal occupation | Not working (housewife); Agriculture; Working paid | Based on the response of respondents, not working women are housewives (usually husbands of such women have paid jobs), agriculture (family's main source of income is agriculture), and paid job |
| Perceived violence | No; Yes | Yes: if women perceived beating in any one of the following conditions: the wife goes out without telling the husband, wife neglects the children, wife argues with husband, wife refuses to have sex with husband; wife burns the food. Otherwise, no perceived violence |
| Household head | Male; Female | This is the decision-maker in the family, as an indicator of women's empowerment |
| Decision-making authority | No; Yes | Whether women participated in at least one of the decisions regarding their health care, purchases or visits to their family or relatives' households |
| **Intermediary** |  |  |
| Provinces | One, Two, Bagmati, Gandaki, Lumbini, Karnali, Sudurpaschim | Provincial assembly of provinces 1 and 2 has yet to decide name of respective province. |
| Languages | Nepali, Maithili, Bhojpuri, and Others (e.g., Tharu, Newari) | The primary language of the respondents |
| Maternal age (in years) | 15-19, 20-34, 35 and above | 15-19, and 35+ years are more at-risk groups |
| Residence | Urban; Rural | Municipalities are called urban, and remaining parts are called rural areas—this rural-urban categorizationis based on socioeconomic indicators of the population. Municipalities have a higher population and development indicators. However, many municipalities which are considered urban areas do not have adequate development facilities |
| Region | Mountain; Hills; Terai | Ecological region |
| Birth order | Less than 4; 4 or more | Numbers of children in the family. |
| Sex of index child | Male; Female | Sex of last birth child |
| Access to bank account | No; Yes | This is a marker of financial empowerment and access to financial resources |
| Media exposure | No; Yes | Received health-related message from at least one of the following once a week: newspaper, radio, or television |
| Last birth (index child) | Unwanted; Wanted | Women perceived the youngest child intentional or not |
| Distance to health facilities is a perceived problem | No problem; a big problem | Perception of problem associated with distance to HF for medical care |
| **Health system** |  |  |
| Perceived problem not having female providers | No problem, big problem | Perceived problem, or not; if no female health provider for healthcare service delivery |
| Awareness on health mothers' group | No; Yes | Awareness of health mothers' groups in the respective wards |
| Mode of delivery | Normal; C-section | Types of childbirth services received by women at health facility |
| **Outcome variable** | | |
| (Dis)continuity of care of MCVs | Had no or at least one to three ANC visits; Had 4ANC visits but no ID; Had 4ANC visits and ID but no PNC visit; Had 4ANC visits and ID and PNC visit | (Dis)continuity of care from the antenatal- postnatal period |

Table S2: Characteristics of women who had a live birth in the two years preceding the survey in Nepal in NDHS 2016.

| **Correlates** | **Categories** | **Frequency** (**N=1978) (%)** |
| --- | --- | --- |
| **Structural** |  |  |
| Wealth status | Lower (40%) | 832 (42.0) |
|  | Upper (60%) | 1146 (58.0) |
| Ethnicity | Disadvantaged | 1374 (69.5) |
|  | Advantaged | 604 (30.5) |
| Religion | Others | 306 (15.5) |
|  | Hindu | 1672 (84.5) |
| Maternal education | No | 570 (28.8) |
|  | Primary | 391 (19.8) |
|  | Secondary or higher | 1016 (51.4) |
| Maternal occupation | Not working | 928 (46.9) |
|  | Agriculture | 824 (41.6) |
|  | Working paid | 227 (11.5) |
| Perceived violence | No | 1397 (70.6) |
|  | Yes | 581 (29.4) |
| Decision-making authority | No | 1324 (66.9) |
|  | Yes | 654 (33.0) |
| Household head | Male | 1438 (72.7) |
|  | Female | 540 (27.3) |
| **Intermediary** |  |  |
| Languages | Nepali | 839 (42.4) |
|  | Maithili | 360 (18.2) |
|  | Bhojpuri | 267 (13.5) |
|  | Others | 512 (25.9) |
| Residence | Urban | 1062 (53.7) |
|  | Rural | 916 (46.3) |
| Province | One | 338 (17.1) |
|  | Two | 513 (25.9) |
|  | Bagmati | 312 (15.8) |
|  | Gandaki | 164 (8.3) |
|  | Lumbini | 364 (18.4) |
|  | Karnali | 121 (6.1) |
|  | Sudurpaschim | 166 (8.4) |
| Region | Mountain | 131 (6.6) |
|  | Hills | 760 (38.4) |
|  | Terai | 1087 (55.0) |
| Maternal age (years) | 15-19 | 291 (14.7) |
|  | 20-34 | 1570 (79.7) |
|  | 35+ | 106 (5.3) |
| Birth order (index child) | <4 | 1678 (84.8) |
|  | ≥4 | 300 (15.2) |
| Sex of index child | Male | 1063 (53.7) |
|  | Female | 915 (46.2) |
| Access to bank account | No | 1367 (69.1) |
|  | Yes | 611 (30.9) |
| Media exposure | No | 911 (46.0) |
|  | Yes | 1067 (54.0) |
| Last birth (index child) | Unwanted | 418 (21.1) |
|  | Wanted | 1560 (78.8) |
| Distance to HFs as a perceived problem | No problem | 763 (38.6) |
|  | Big problem | 1213 (61.4) |
| **Health system** |  |  |
| Perceived problem not having female providers | No problem | 562 (28.4) |
|  | Big problem | 1416 (71.6) |
| Awareness on health mothers' group | No | 1340 (67.7) |
|  | Yes | 638 (32.3) |
| Mode of delivery | Normal | 1780 (90.0) |
|  | Caesarean section | 198 (10.0) |

Table S3: Bivariable multinomial logistic regression analysis of the (dis)continuity of routine MCVs in Nepal, NDHS 2016.

| **Correlates** | **Categories** | **Had no or <4ANC visits (vs all three visits)**  **Unadjusted RR (95% CI)** | **Had ≥4ANC visits but no ID (vs all three visits) Unadjusted RR (95% CI)** | **Had ≥4ANC visits and ID but no PNC visit (vs all three visits) Unadjusted RR (95% CI)** |
| --- | --- | --- | --- | --- |
| **Structural** |  |  |  |  |
| Wealth rank | Upper | 1.00 | 1.00 | 1.00 |
|  | Lower (60%) | 2.27(1.69, 3.07) *** | 3.33(2.39,4.63) *** | 0.97(0.66, 1.42) |
| Ethnicity | Advantaged | 1.00 | 1.00 | 1.00 |
|  | Disadvantaged | 3.01(2.08, 4.35) *** | 1.96(1.40, 2.73) *** | 1.30(0.92, 1.83) |
| Religion | Other | 1.00 | 1.00 | 1.00 |
|  | Hindu | 0.58(0.38, 0.89) * | 0.96(0.61, 1.51) | 1.00 (0.59, 1.68) |
| Maternal occupation | Agriculture | 1.00 | 1.00 | 1.00 |
|  | Housewife | 1.10(0.83, 1.45) | 0.50(0.37, 0.68) *** | 1.19(0.80, 1.76) |
|  | Working paid | 0.49(0.30, 0.80) ** | 0.38(0.23, 0.64) *** | 1.02(0.58, 1.79) |
| Maternal education | Higher | 1.00 | 1.00 | 1.00 |
|  | Illiterate | 6.16 (4.38, 8.65) *** | 3.00(2.11, 4.25) *** | 1.34(0.86, 2.08) |
|  | Primary | 4.15 (2.94, 5.87) *** | 3.18(2.17, 4.67) *** | 1.32(0.83, 2.08) |
| Perceived violence | No | 1.00 | 1.00 | 1.00 |
|  | Yes | 1.43(1.07, 1.91) * | 1.28(0.92, 1.79) | 1.51(1.06, 2.15) * |
| Decision making | Yes | 1.00 | 1.00 | 1.00 |
|  | No | 1.39(1.06, 1.83) * | 1.26(0.89, 1.80) | 1.06(0.76, 1.49) |
| Household head | Male | 1.00 | 1.00 | 1.00 |
|  | Female | 0.72 (0.55, 0.93) * | 0.76(0.55, 1.04) | 0.77(0.52, 1.15) |
| **Intermediary** |  |  |  |  |
| Language | Nepali | 1.00 | 1.00 | 1.00 |
|  | Maithili | 2.52(1.61, 3.96) *** | 2.11(1.29, 3.43) ** | 1.30(0.81, 2.08) |
|  | Bhojpuri | 6.36(3.84, 10.55) *** | 1.57(0.81, 3.05) | 2.13(1.06, 4.26) * |
|  | Others | 1.70 (1.16, 2.49) ** | 1.18(0.80, 1.72) | 0.89(0.60, 1.31) |
| Province | One | 1.00 | 1.00 | 1.00 |
|  | Two | 3.50(2.09, 5.84) *** | 1.47(0.84, 2.59) | 2.56(1.32, 4.96) ** |
|  | Bagmati | 1.07(0.56, 2.02) | 0.58(0.29, 1.15) | 1.20(0.61, 2.36) |
|  | Gandaki | 1.02(0.57 ,1.81) | 0.45(0.22, 0.90) * | 0.95(0.46, 1.96) |
|  | Lumbini | 1.36(0.73, 2.52) | 0.97(0.59, 1.60) | 1.86(0.97, 3.58) |
|  | Karnali | 4.30(2.55, 7.25) *** | 1.93(1.04, 3.58) * | 2.19(1.07, 4.47) * |
|  | Sudurpaschim | 0.79(0.47, 1.32) | 0.70(0.37, 1.33) | 2.89(1.51, 5.54) ** |
| Region | Hill | 1.00 | 1.00 | 1.00 |
|  | Mountain | 1.46(0.72, 2.97) | 2.05(1.04, 4.02) * | 0.35(0.11, 1.10) |
|  | Terai | 1.74(1.22, 2.48) ** | 1.17(0.82, 1.66) | 1.40(0.98, 2.00) |
| Residence | Urban | 1.00 | 1.00 | 1.00 |
|  | Rural | 2.12 (1.48 ,3.04) *** | 2.51(1.76,3.59) *** | 1.12(0.77,1.61) |
| Maternal age (in years) | 15-19 | 1.00 | 1.00 | 1.00 |
|  | 20-34 | 1.15(0.76, 1.74) | 1.22(0.80, 1.86) | 0.67(0.44, 1.01) |
|  | 35 above | 1.88(0.96, 3.66) | 1.62(0.69, 3.80) | 0.78(0.32, 1.91) |
| Birth order | <4 | 1.00 | 1.00 | 1.00 |
|  | ≥4 | 4.54(3.09, 6.68) *** | 2.70(1.88, 3.89) *** | 0.71(0.38, 1.34) |
| Sex of index child | Male | 1.00 | 1.00 | 1.00 |
|  | Female | 1.08(0.85, 1.37) | 1.18(0.88, 1.58) | 1.02(0.75, 1.38) |
| Access to bank account | Yes | 1.00 | 1.00 | 1.00 |
|  | No | 2.89(2.02, 4.14) *** | 2.23(1.62, 3.07) *** | 1.44(1.01, 2.06) * |
| Media exposure | Yes | 1.00 | 1.00 | 1.00 |
|  | No | 3.72 (2.85, 4.85) *** | 2.83(2.12, 3.79) *** | 1.27(0.90, 1.78) |
| Last birth (index child) | Wanted |  |  |  |
|  | Unwanted | 1.95(1.38, 2.76) *** | 1.12(0.76, 1.66) | 1.18(0.75, 1.87) |
| Distance to health facilities was a perceived problem | No problem | 1.00 | 1.00 | 1.00 |
|  | Big problem | 2.17(1.64, 2.89) *** | 2.39(1.75, 3.25) *** | 1.47(1.03, 2.08) * |
| **Health system** |  |  |  |  |
| Perceived problem not having female providers | No problem | 1.00 | 1.00 | 1.00 |
|  | Big problem | 2.16(1.56, 2.99) *** | 2.13(1.50, 3.02) *** | 1.74(1.23, 2.46) ** |
| Awareness on health mothers' group | Yes | 1.00 | 1.00 | 1.00 |
|  | No | 1.75(1.36, 2.25) *** | 1.06(0.79, 1.41) | 1.07(0.77, 1.48) |
| Mode of delivery | C-section | 1.00 | - | 1.00 |
|  | Normal delivery | 3.62(2.04, 6.45) *** | - | 1.73(1.06, 2.84) * |
